# Exosomes derived from M2 type tumor-associated macrophages promote osimertinib resistance in non-small cell lung cancer through MSTRG.292666.16-miR-6836-5p-MAPK8IP3 axis

**DOI:** 10.1186/s12935-022-02509-x

**Published:** 2022-02-15

**Authors:** Xiaoying Wan, Boxiong Xie, Hui Sun, Weiqing Gu, Chunyan Wang, Qinfang Deng, Songwen Zhou

**Affiliations:** 1 Department of Medical Oncology, Shanghai Pulmonary Hospital, Tongji University School of Medicine, No.507, Zhengmin Road, Yangpu District, Shanghai, 200433 China; 2 Medical College of Tongji University, Shanghai, 200433 China; 3 Department of Thoracic, Shanghai Pulmonary Hospital, Tongji University School of Medicine, Shanghai, 200433 China

**Keywords:** NSCLC, M2-type TAM, Exosomes, Osimertinib resistance, MSTRG.292666.16, miR-6836-5p, MAPK8IP3

## Abstract

**Background:**

Osimertinib resistance limits the treatment of epidermal growth factor receptor-(EGFR)-mutated non-small-cell lung carcinoma (NSCLC). The mechanisms of osimertinib resistance need to be elucidated to determine alternative treatment strategies. This study explores the role of M2 type tumor-associated macrophage (TAM)-derived exosomal MSTRG.292666.16 in osimertinib resistance, and its related competing endogenous RNA (ceRNA) mechanism.

**Methods:**

M2 type TAMs were induced with 200 ng/mL phorbol 12-myristate 13-acetate, 20 ng/mL IL-4 and IL-13, and M2 type macrophage markers were measured by RT-qPCR. Next, the exosomes were isolated and characterized. Tumor formation in nude mice was conducted using H1975 cells under different treatment conditions. Small RNA sequencing was performed on exosomes derived from sensitive and resistant plasma, and ceRNA networks were constructed. Fluorescence in situ hybridization was used to observe the localization of MSTRG.292666.16, and a ceRNA network (MSTRG.292666.16-miR-6836-5p-MAPK8IP3) was selected for further validation.

**Results:**

M2 type TAMs, and M2 type TAM-derived exosomes were successfully induced and isolated. Nude mice results showed that M2 type TAM-derived exosomes and MSTRG.292666.16 overexpression significantly increased tumor volume after administration of osimertinib for 4 weeks. M2 type TAMs were found in the resistant plasma, and MSTRG.292666.16 localized in the cytoplasm of H1975 cells. In addition, the genes in the ceRNA networks were significantly enriched in eight GO terms and seven KEGG pathways, including the MAPK signaling pathway. Subsequently, the levels of MSTRG.292666.16 and MAPK8IP3 significantly increased in both resistant plasma-derived exosomes and M2 type TAM-derived exosomes, while miR-6836-5p levels were significantly reduced. Finally, MSTRG.292666.16, miR-6836-5p, and MAPK8IP3 were part of the same network.

**Conclusions:**

M2 type TAM-derived exosomes promoted osimertinib resistance in NSCLC by regulating the MSTRG.292666.16/miR-6386-5p/MAPK8IP3 axis.

**Supplementary Information:**

The online version contains supplementary material available at 10.1186/s12935-022-02509-x.

## Background

Lung cancer is the most common cancer worldwide and has the highest mortality rate [1]. Non-small cell lung cancer (NSCLC) accounts for 85% of all cases of lung cancer, with a 5-year survival rate of only 21% [2]. Currently, surgery, chemotherapy, and radiation are the traditional treatments for lung cancer; however or patients with advanced disease, surgery is not sufficient to restrain cancer progression [3]. With the continuous update of gene detection technology, epidermal growth factor receptor-tyrosine kinase inhibitors (EGFR-TKIs) have become an essential method for precision treatment of NSCLC [4]. Osimertinib, a third-generation EGFR-TKI, selectively and irreversibly targets EGFR mutations (e.g., T790M [c.2369 C > T; p.Thr790Met], L858R [c.2572-2573 inseAG, c.2573T > G, c.2573delinsGA, and c.2574delinsGT; p.Leu858Arg], and C797S [c.2389T > A and c.2390G > C; p.Cys797Ser]), while sparing the wild-type EGFR tyrosine kinase [5]. Recent studies have reported that the overall survival of patients with EGFR-mutated advanced NSCLC was up to 38.6 months after osimertinib treatment, and postoperative adjuvant therapy with osimertinib significantly increased disease-free survival [6, 7]. Therefore, osimertinib is a powerful tool in the first-line treatment of EGFR-mutated advanced NSCLC and in adjuvant therapy for NSCLC after surgery. However, osimertinib resistance is a major obstacle in the treatment of NSCLC, and the resistance mechanisms have not been fully elucidated.

Exosomes are nanoscale lipid inclusions that can carry RNA and proteins and are an important system for intercellular signal transduction and substance delivery [8]. Previous studies have indicated that exosomes participate in various process of tumor drug resistance through the transmission of long non-coding RNA (lncRNA), microRNA (miRNA), and mRNA [9, 10]. Macrophages are one of the major populations of tumor-infiltrating immune cells, and tumor-associated macrophages (TAMs) are the primary actors and coordinators of tumor progression in the microenvironment [11]. Macrophages are usually polarized to either M1 or M2 type; while M1 type macrophages have pro-inflammatory properties, M2 type macrophages contribute to anti-inflammatory response and immune homeostasis [12]. M2 type TAMs secrete a variety of cytokines, chemokines, and exosomes, thus promoting invasion and chemoresistance of ovarian cancer cells [13], as well as transfer miR-21 to gastric cancer cells thus conferring cisplatin resistance [14]. However, the effects of M2 type TAM-derived exosomes on osimertinib resistance in NSCLC remain unknown.

Competing endogenous RNA (ceRNA) can regulate the expression of downstream mRNAs by competing with shared miRNAs, and an imbalance of ceRNA networks influences cell processes and functions, thus leading to the occurrence and development of diseases [15, 16]. In ceRNA networks, lncRNAs, serving as ceRNAs, can participate in the initiation and progression of liver cancer by sponging miRNA response elements. A study by Xu et al. found that lncRNA MIR194-2HG was highly expressed in liver cancer samples and activated the Wnt/β-catenin signaling pathway through the miR-1207-5p/TCF19 axis, thereby playing important roles in the diagnosis and treatment of liver cancer [17]. Our previous study showed that the level of lncRNA MSTRG.292666.16 was significantly higher in the osimertinib-resistant plasma-derived exosomes than in sensitive plasma-derived exosomes, and found that exosomal lncRNA MSTRG.292666.16 could promote acquired resistance to osimertinib and play a key role in drug resistance transmission [18]. However, the ceRNA mechanisms of exosomal lncRNA MSTRG.292666.16 in osimertinib resistance in NSCLC are still unclear.

Therefore, in this study, we further confirmed the osimertinib resistance of M2 type TAM-derived exosomal lncRNA MSTRG.292666.16 to NSCLC in vivo. Moreover, small RNA sequencing of exosomes was performed and combined with the previous lncRNA sequencing data. Furthermore, the ceRNA mechanism of lncRNA MSTRG.292666.16 in NSCLC osimertinib resistance was investigated, and a ceRNA network of lncRNA MSTRG.292666.16-hsa-miR-6836-5p-MAPK8IP3 was selected for further validation. These results would provide new insights into the development of novel therapeutic targets and pathways to overcome osimertinib-acquired resistance in NSCLC.

## Materials and methods

### Patients

The patient samples were collected based on a previous study [18] with some minor modifications. Briefly, from October 2015 to September 2018, seven NSCLC patients were recruited from the Department of Oncology, Shanghai Pulmonary Hospital. The clinical characteristics of these patients are shown in Table 1. These patients were administrated first-generation EGFR-TKIs. However, after the development of acquired resistance, osimertinib was used to treat these patients for approximately nine months. Among them, four patients developed osimertinib acquired resistance, and three patients remained sensitive to osimertinib. After that, the patients were divided into sensitive (n = 3) and resistant (n = 4) groups, and the plasma of each patient was obtained for exosome isolation. The study was approved by the Ethics Committee of Shanghai Pulmonary Hospital, and informed consent was obtained from all participants.

**Table 1:** The clinical information of the obtained patients treated with osimertinib for about 9 months

No	Sex	Age	Disease	Distant metastases	Smoke	Resistance to osimertinib
1	Male	56	Left upper-lobe adenocarcinoma	Yes	Yes	Resistant
2	Female	61	Right lower-lobe adenocarcinoma	Yes	No	Resistant
3	Female	53	Right middle-lobe adenocarcinoma	Yes	No	Resistant
4	Female	64	Left upper-lobe adenocarcinoma	Yes	No	Resistant
5	Male	72	Left lower-lobe adenocarcinoma	Yes	Yes	Sensitive
6	Female	65	Left upper-lobe adenocarcinoma	Yes	No	Sensitive
7	Male	57	Left upper-lobe adenocarcinoma	Yes	Yes	Sensitive

### Cell culture and induction of M2 type TAM

Human lung cancer H1975 cells and THP-1 cells were obtained from the Cell Bank of the Chinese Academy of Sciences (Shanghai, China). The H1975 cells were cultured in Dulbecco’s modified of Eagle’s medium (DMEM, Gibco, Grand Island, NY) supplemented with 10% fetal bovine serum (FBS, Gibco) and 1% penicillin/streptomycin (Gibco), and incubated in an incubator with 5% CO_2_ at 37 °C. THP-1 cells were maintained in RPMI1640 medium (Gibco) with 10% FBS, and then used to induce the formation of M2 type TAM as previously described [19]. Briefly, THP-1 cells were divided into two groups: control and M2-TAM groups. The cells in the control group were cultured in RPMI1640 medium with 10% FBS. However, the cells in the M2-TAM group were incubated in medium containing 200 ng/mL phorbol 12-myristate 13-acetate (PMA, Sigma) for 24 h. Then, 20 ng/mL interleukin-4 (IL-4, PeproTech) and 20 ng/mL IL-13 (PeproTech) were added to the medium and cultured for another 72 h. Thereafter, the cells were harvested to determine the levels of M2 type macrophage markers (CD206, CD163, TGF-β, IL-10, and Arg-1) using reverse-transcription quantitative PCR (RT-qPCR). The successfully constructed cells were then used for exosome isolation.

### Isolation and characterization of exosomes from the plasma and M2 type TAMs

The exosomes were isolated from the plasma and M2 type TAMs using differential centrifugation under temperature conditions at 4 °C, as previously reported [20]. The plasma was sequentially centrifuged at 500×*g* for 10 min, 2000×*g* for 30 min, and 10,000×*g* for 30 min. The supernatant was filtered using 0.22 μm filters (Millipore, USA), followed by centrifugation at 120,000×*g* for 70 min. The sediment, including the exosomes, was resuspended in 200 μL of PBS. For the isolation from the M2 type TAMs, the cells were centrifuged at 300×*g* for 10 min, 10,000×*g* for 30 min, and 100,000×*g* for 70 min. The sediment was resuspended in 1 mL PBS, and then centrifuged at 10,000×*g* for 60 min. The exosomes were resuspended in 200 μL PBS and stored at −80 °C until use.

The concentrations of the isolated exosomes were measured using a BCA protein assay kit (BOSTER Biological Technology Co. Ltd, CA, USA). Then, a NanoSight NS300 particle size analyzer (NTA, Malvern Panalytical, Malvern, UK) was used to analyze the particle size of the exosomes based on the methods described by Soares et al. [21]. According to the protocols of Zhu et al. [22], the morphology of the exosomes was visualized using transmission electron microscopy (TEM, JEOL LTD, Peabody, MA, USA). Finally, western blotting was used to determine the expression of exosome-specific markers (CD9, HSP70, and TSG101) with their corresponding antibodies (1:1000, Proteintech Group, Inc., Rosemont, IL, USA) as previously described [23].

### Small RNA sequencing of plasma-derived exosomes

The exosomes isolated from the plasma prior to osimertinib treatment (sensitive group) and their corresponding drug-resistant plasma (resistant group) were delivered to Yanzai Biotechnology (Shanghai) Co. Ltd (Shanghai, China) for small RNA sequencing [24]. The clean reads were aligned to the databases of human miRNA, genome, and Rfam, and Burrows–Wheeler Aligner (BWA) mapping software was used to analyze the gene expression profiles. Next, the DESeq algorithm was applied to screen the differentially expressed miRNAs (DEmiRNAs) with the thresholds of |log_2_Fold change (FC)|> 1 and false discovery rate (FDR) < 0.05. Then, the Miranda and RNAhybrid algorithms (http://www.microrna.org) were used to predict the target genes of the identified DEmiRNAs. Afterwards, these screened DEmiRNAs were submitted for Gene Ontology (GO) and Kyoto Encyclopedia of Genes and Genomes (KEGG) pathway analyses. *P* < 0.05 was considered as the criterion for significantly enriched GO terms and KEGG pathways.

### Construction of ceRNA networks

A previous study by Deng et al. [18] reported 123 differentially expressed lncRNAs (DElncRNAs) between exosomes isolated from the plasma of sensitive and resistant groups through lncRNA sequencing. Here, we combined the sequencing results of lncRNAs and miRNAs to construct ceRNA networks. First, an online tool miRanda (http://cbio.mskcc.org/miRNA2003/miranda.html) was used to screen the lncRNA-miRNA pair. Subsequently, the RNAhybrid (http://bibiserv.techfak.uni-bielefeld.de/rnahybrid/) and miRanda (http://www.microrna.org/microrna/home.do) databases were used to predict the target genes of the aforementioned DEmiRNAs screened from the lncRNA-miRNA co-expression pairs, and the co-expression pairs of miRNA-mRNA with the opposite expression direction were retained. After that, ceRNA networks were established using lncRNA-miRNA-mRNA co-expression pairs and visualized using Cytoscape (version 3.7.0). Because exosomal lncRNA MSTRG.292666.16 has been reported to be associated with osimertinib resistance in NSCLC [18], we chose a ceRNA relationship of lncRNA MSTRG.292666.16 -miRNA hsa-miR-6836-5p-mRNA MAPK8IP3 for further experiments.

### *Fluorescence *in Situ* Hybridization (FISH)*

The localization of lncRNA MSTRG.292666.16 in H1975 cells was determined using a FISH assay kit (Guangzhou RiboBio Co., Ltd, Guangzhou, China) following the manufacturer’s protocols. The H1975 cells were seeded in a 24-well plate at a density of 5 × 10^4^ cells/well and cultured to a confluence of approximately 70%. The cells were fixed with 4% paraformaldehyde for 10 min at room temperature (RT). After washing with PBS, 1 mL of pre-cooled transparent liquid (PBS containing 0.5% Triton X-100) was added to each well and incubated at 4 °C for 5 min. After removing the transparent liquid and a washing step, a prehybridization solution (200 μL) was added and blocked at 37 °C for 30 min. After removing the prehybridization solution, 2.5 μL of 20 μM lncRNA MSTRG.292666.16 FISH probe were mixed into 100 μL hybrid solution, the mixture was added to the cells and incubated in the dark at 37 °C overnight. In the dark at 42 °C, the cells were washed with hybridization solutions I, II, III, and PBS. Finally, the cells were sealed with DAPI, and a fluorescence microscope (IX70, Olympus Corporation, Japan) was used to observe the images.

### Cell transfection

Our previous in vitro study found that exosomal lncRNA MSTRG.292666.16 was closely associated with osimertinib resistance in NSCLC. Therefore, in this study, we mainly explored its roles in vivo. We first constructed H1975 cells overexpressing lncRNA MSTRG.292666.16, as previously described [25]. Briefly, the pCDH vector and pCDH-MSTRG.292666.16 were prepared and provided by Yanzai Biotechnology (Shanghai) Co. Ltd. The H1975 cells were seeded into a 6-well plate at a density of 4 × 10^5^ cells/well and cultured overnight. The next day, the medium was changed to serum-free medium, and the cells were transfected with 3 μg pCDH vector and 3 μg pCDH-MSTRG.292666.16 using Lipofectamine 3000 (Thermo Fisher Scientific, Waltham, MA, USA) according to the manufacturer’s instructions. After transfected for 6 h, the medium was replaced with complete medium and cultured for another 48. The level of MSTRG.292666.16 in the cells with different treatments was measured by RT-qPCR to evaluate the cell transfection efficiency. The sequences of MSTRG.292666.16 are shown in Table 2.

**Table 2:** The sequences of all primers

Primer		Sequence (5’-3’)
MSTRG.292666.16	F	CTGGAGTGCAGTGGCTATTC
	R	AGGCTGAGGTGGGAGGAT
MAPK8IP3	F	GTGTACCAGGACGACTACTGC
	R	GCACCGAGTCTAGGTTCTCCA
CD206	F	TCCGGGTGCTGTTCTCCTA
	R	CCAGTCTGTTTTTGATGGCACT
CD163	F	TTTGTCAACTTGAGTCCCTTCAC
	R	TCCCGCTACACTTGTTTTCAC
TGF-β	F	GGCCAGATCCTGTCCAAGC
	R	GTGGGTTTCCACCATTAGCAC
IL-10	F	GACTTTAAGGGTTACCTGGGTTG
	R	TCACATGCGCCTTGATGTCTG
Arg-1	F	GTGGAAACTTGCATGGACAAC
	R	AATCCTGGCACATCGGGAATC
GAPDH	F	TGACAACTTTGGTATCGTGGAAGG
	R	AGGCAGGGATGATGTTCTGGAGAG
miR-6836-5p	R	GTCGTATCCAGTGCAGGGTCCGAGGTATTCGCACTGGATACGACATGCCT
	F	CGCAGGGCCCTGGCGC
U6	R	AACGCTTCACGAATTTGCGT
	F	CTCGCTTCGGCAGCACA
Universal downstream primer		GTGCAGGGTCCGAGGT

### Dual-luciferase reporter gene assay

The sequences of WT/MUT MSTRG.292666.16 lncRNA inserts, miR-6836-5p mimics/negative control (NC) mimics, and WT/MUT MAPK8IP3 3′-untranslated regions (3′-UTRs) were synthesized and purchased from Yanzai Biotechnology (Shanghai) Co. Ltd. The psiCHECK2 vector (Yanzai Biotechnology (Shanghai) Co. Ltd) and pGL3 vector (Yanzai biotechnology (Shanghai) Co. Ltd) were used to generate the psiCHECK-MSTRG.292666.16 reporter plasmid (psiCHECK-MSTRG.292666.16 WT/MUT) and the 3′-UTR MAPK8IP3 reporter plasmid (pGL3-MAPK8IP3 WT/MUT), respectively. 293 T cells (Cell Bank, Chinese Academy of Sciences) were seeded into a 96-well plate and cultured overnight. The following day, the psiCHECK2-MSTRG.292666.16 WT/MUT (0.3 μg) or psiCHECK vector (0.3 μg), and pGL3-MAPK8IP3 WT/MUT (0.3 μg) or pGL3 vector (0.3 μg) were co-transfected into 293 T cells with NC mimics (100 nM) or miR-6836-5p mimics (100 nM) using Lipofectamine 3000 (Thermo Fisher Scientific) based on the manufacturer’s recommendations. After transfection for 6 h and culture for another 48 h, the cells were harvested to determine luciferase activity using a dual luciferase reporter system (Promega, Madison, WI, USA).

### RT-qPCR

Total RNA was isolated from the plasma and M2 type TAMs using the mirVANA miRNA Isolation kit (Thermo Fisher Scientific) according to the manufacturer’s instructions. The quality and concentration of total RNA was assessed using a microplate reader. Total RNA was reverse transcribed into cDNA using the PrimeScriptTM II 1st Strand cDNA synthesis Kit (Takara Biomedical Technology Co., Ltd., Beijing, China), according to the manufacturer’s protocols. For miRNA, the stem loop RT-qPCR method was used, and U6 was used as a reference gene [26]. For lncRNA and mRNA, glyceraldehyde-3-phosphate dehydrogenase (*GAPDH*) served as a housekeeping gene. The RT-qPCR reaction was initiated at 95 °C for 3 min, followed by 40 cycles of 95 °C for 10 s and 60 °C for 30 s. The levels of lncRNA MSTRG.292666.16, miR-6836-5p, MAPK8IP3, and TAM-related markers (*CD206*, *CD163*, *TGF-β*, *IL-10*, and *Arg-1*) were calculated using the 2^−ΔΔCt^ method. The sequences of all primers are listed in Table 2.

### Animal experiments

Before animal experiments, H1975 cells were divided into four groups: control, H1975 + M2 type TAM-derived exosomes, H1975 + pCDH, and H1975 + pCDH-lncRNA groups. The cells in the H1975 + M2 type TAM-derived exosomes group were treated with 10 μg/mL M2 type TAM-derived exosomes, and the cells in the H1975 + pCDH and H1975 + pCDH-lncRNA groups were transfected with pCDH and pCDH-lncRNA, respectively. No treatment was applied for the cells in the control group. After that, the medium was removed, and the final concentration of cells in the different groups was adjusted to 2 × 10^7^ cells/mL with PBS.

Twenty SPF BALB/C female nude mice aged 4–6 weeks were purchased from Shanghai SLAC Laboratory Animal Co., Ltd. (Shanghai, China). All mice were maintained under controlled temperature (24 ± 2 °C) and humidity (50 ± 5%) conditions, with a 12 h light/dark cycle. The rats were given ad libitum access to food and water during the experiment. After 7 days of acclimatization, the mice were randomly divided into four groups (n = 5 for each group): control, M2 TAM-derived Exos, pCDH, and pCDH-lncRNA groups. The mice in the M2 TAM-derived Exos, pCDH, and pCDH-lncRNA groups were subcutaneously injected with 0.1 mL H1975 cells pre-treated with M2 type TAM-derived exosomes (2 × 10^7^ cells/mL), 0.1 mL H1975 cells transfected with pCDH (2 × 10^7^ cells/mL) and 0.1 mL H1975 cells transfected with pCDH-lncRNA (2 × 10^7^ cells/mL), respectively. The mice in the control group were injected with PBS. On the 10th day of modeling, osimertinib (2.5 mg/kg/d) was intraperitoneally injected into each mouse for four weeks. The long and short diameters of the tumors were measured weekly to calculate the tumor volume. After four weeks, the mice were sacrificed by cervical dislocation, and the tumor was removed, weighed, and photographed. All animal experiments were performed in accordance with the National Medical Advisory Committee (NMAC) guidelines using approved procedures of the Institutional Animal Care and Use Committee at Shanghai Pulmonary Hospital.

### Statistical analysis

Data are expressed as mean ± standard deviation (SD), and GraphPad Prism 5 (GraphPad Software, CA, USA) was used for all statistical analyses. Student’s *t*-test was used for comparisons between the two groups. One-way analysis of variance (ANOVA) followed by Tukey’s test was used to compare the differences among more than two groups. Statistical significance was set at *P* < 0.05.

## Results

### Induction of M2 phenotype in TAM

**Fig. 1:**
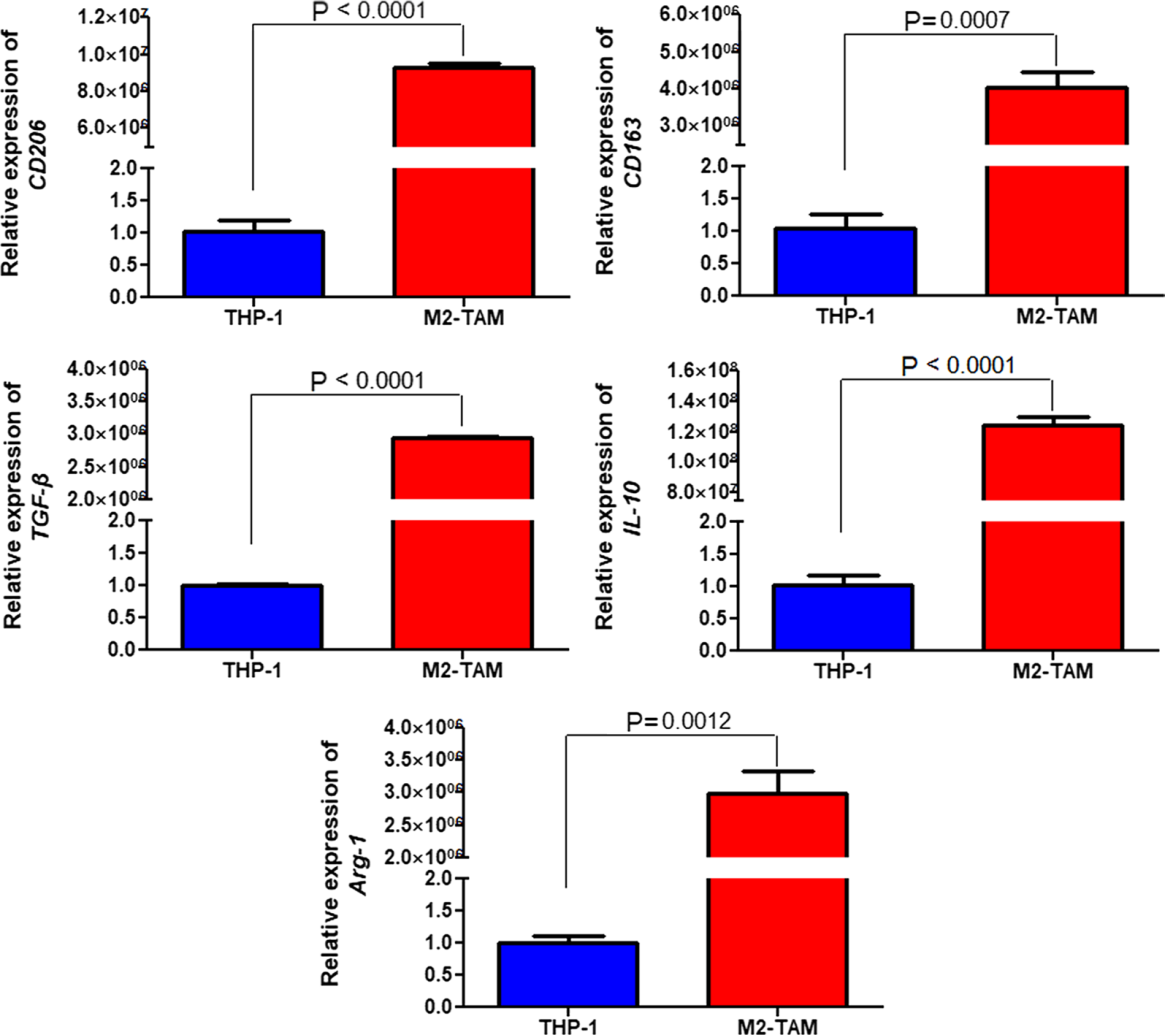
Induction of M2 type tumor-associated macrophages (TAMs). The expression levels of *CD206*, *CD163*, *TGF-β*, *IL-10* and *Arg-1* were significantly increased in the M2 type TAMs compared with the THP-1 cells, which indicated that M2 phenotype in TAMs was successfully induced by PMA, IL-4, and IL-13

PMA, IL-4, and IL-13 were used to induce THP-1 cells to form M2 type TAMs, and the markers of M2 type macrophage (CD206, CD163, TGF-β, IL-10, and Arg-1) were measured. It was found that the expression levels of *CD206*, *CD163*, *TGF-β*, *IL-10*, and *Arg-1* were significantly increased in the M2 type TAMs compared with those in THP-1 cells (*P* < 0.05, Fig. 1), which indicated that M2 type TAMs were successfully induced by PMA, IL-4, and IL-13.

### Characterization of exosomes isolated from M2 type exosomes

**Fig. 2:**
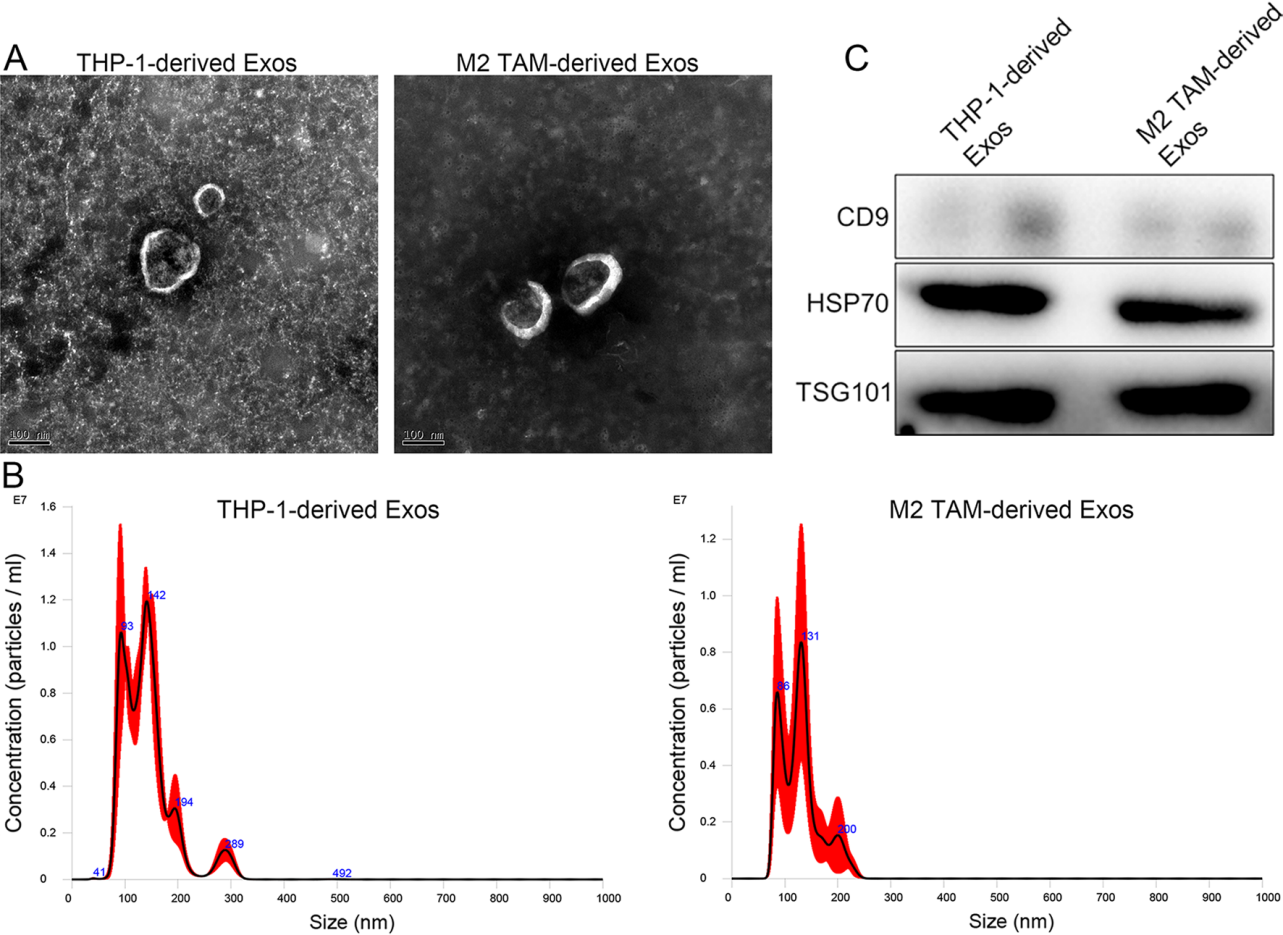
Identification of THP-1-derived and M2 type TAM-derived exosomes. **A** Transmission electron microscopy was used to observe the morphology of exosomes isolated from THP-1 cells and M2 type TAMs. **B** Particle size distributions of THP-1 cells-derived and M2 type TAM-derived exosomes were measured by a Nanosight NS300 particle size analyzer. **C** Exosomes-specific markers (CD9, HSP70 and TSG101) were detected by western blot. All the results indicated that exosomes were successfully isolated from the THP-1 cells and M2 type TAMs

TEM, NTA, and western blot were used to identify the exosomes from THP-1 cells and successfully induced M2 phenotype in TAMs. TEM results showed that the exosomes isolated from the TPH-1 cells and M2 type TAMs were nearly round with a diameter of approximately 100 nm (Fig. 2A). As shown in Fig. 2B, the major peaks of the exosomes isolated from the TPH-1 cells and M2 type TAMs were respectively 142 nm and 131 nm, and their average peaks were respectively 141.1 ± 48.3 nm and 128.6 ± 36.8 nm. These results were in accordance with the size distribution of exosomes, as previously reported [24]. Finally, western blot showed that CD9, HSP70, and TSG101, which are exosome-specific markers, were all expressed in THP-1-derived exosomes and M2 type TAM-derived exosomes (Fig. 2C). These results indicate that exosomes were successfully isolated from THP-1 cells and M2 type TAMs.

### *The effects of M2 type TAM-derived exosomal MSTRG.292666.16 on osimertinib resistance *in vivo

**Fig. 3:**
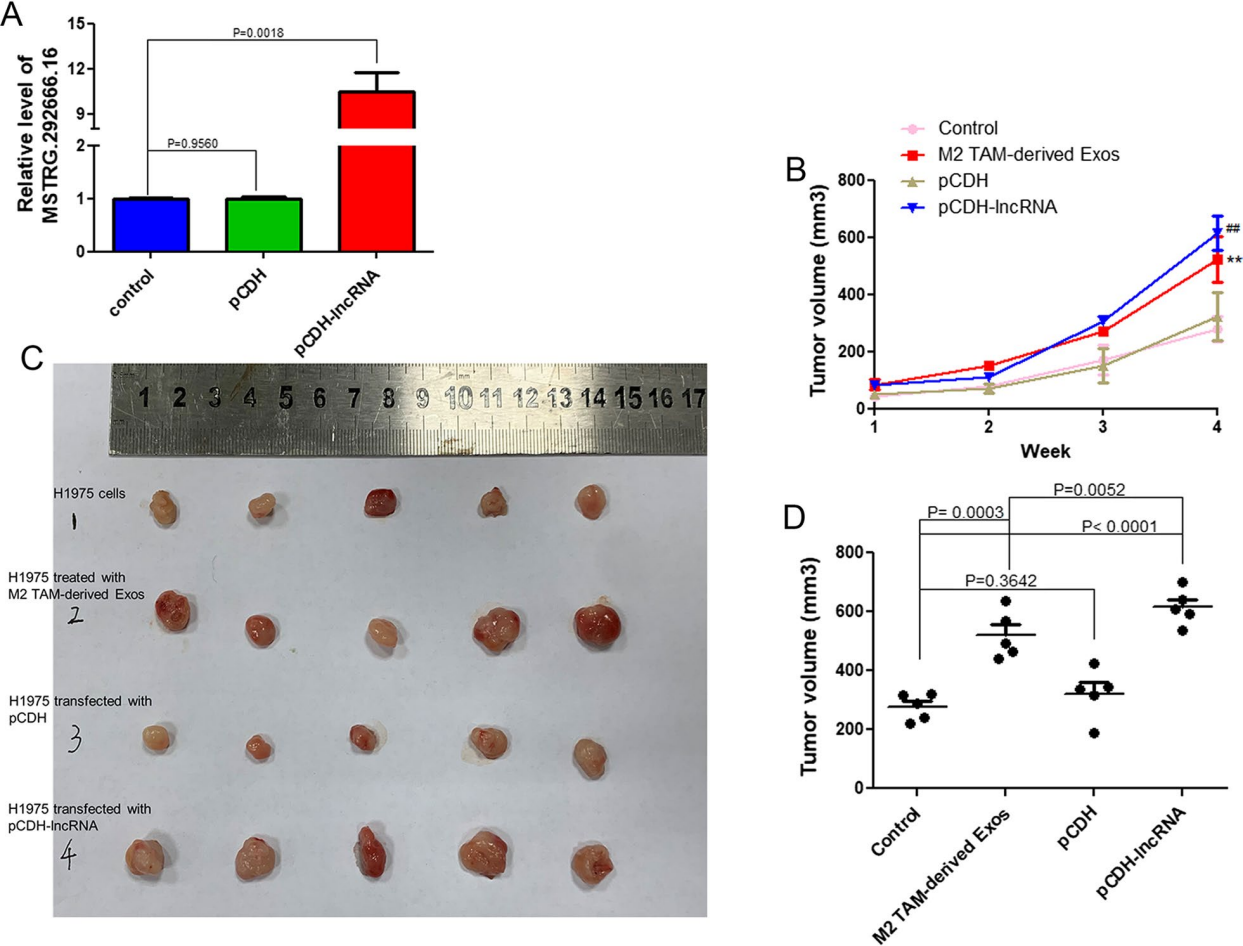
Effects of M2 type TAM-derived exosomal lncRNA MSTRG.292666.16 on osimertinib resistance in non-small cell lung cancer (NSCLC) in vivo. **A** The level of MSTRG.292666.16 in the pCDH-lncRNA group was significantly increased compared with the control group, which suggested that H1975 cells with lncRNA MSTRG.292666.16 overexpression have been successfully established. **B** The changes of tumor volume with the increase of administration time. **: *P* < 0.01, compared with the control group; ^##^: *P* < 0.01, compared with the pCDH group. **C** The size of tumor in different groups after administrated with osimertinib for four weeks. **D** The tumor volume in different groups after four weeks of osimertinib treatment. The in vivo experiments indicated that M2 type TAM-derived exosomes and lncRNA MSTRG.292666.16 overexpression could enhance osimertinib resistance in NSCLC

H1975 cells overexpressing lncRNA MSTRG.292666.16 were constructed to investigate the roles of exosomal lncRNA MSTRG.292666.16 in osimertinib-resistant NSCLC. No significant difference was found in the relative level of lncRNA MSTRG.292666.16 between the control and pCDH groups (*P* = 0.9560 > 0.05, Fig. 3A). The relative level of lncRNA MSTRG.292666.16 in the pCDH-lncRNA group was significantly was higher than that in the control group (*P* = 0.0018, Fig. 3A). These results suggest that H1975 cells overexpressing lncRNA MSTRG.292666.16 have been successfully established. Then, the effects of exosomal MSTRG.292666.16 on osimertinib resistance were determined by subcutaneously injecting the cells with different treatments into nude mice in vivo. After tumor formation, nude mice were administered osimertinib for four weeks. With the increase in time, there was no significant difference in tumor volume between the control and pCDH groups (*P* > 0.05, Fig. 3B). However, the tumor volume in the M2 TAM-derived Exos and pCDH-lncRNA groups significantly increased with time (*P* < 0.05, Fig. 3B). After four weeks, the tumor volumes in the different groups were measured. The tumor volume in the control and pCDH groups was respectively 277.53 ± 43.73 mm^3^ and 322.48 ± 85.21 mm^3^, which showed no significant difference between the two groups (*P* = 0.3642 > 0.05, Fig. 3C, D). Compared with the control group, the tumor volume was evidently increased after M2 type TAM-derived exosomes (*P* = 0.0003 < 0.05) and lncRNA MSTRG.292666.16 overexpression (*P* < 0.0001) pre-treatment (Fig. 3C, D). These in vivo results strongly suggest that M2 type TAM-derived exosomes and lncRNA MSTRG.292666.16 overexpression could enhance osimertinib resistance in NSCLC.

### M2 type TAM-related markers in the plasma, and the localization of MSTRG.292666.16 in cells

**Fig. 4:**
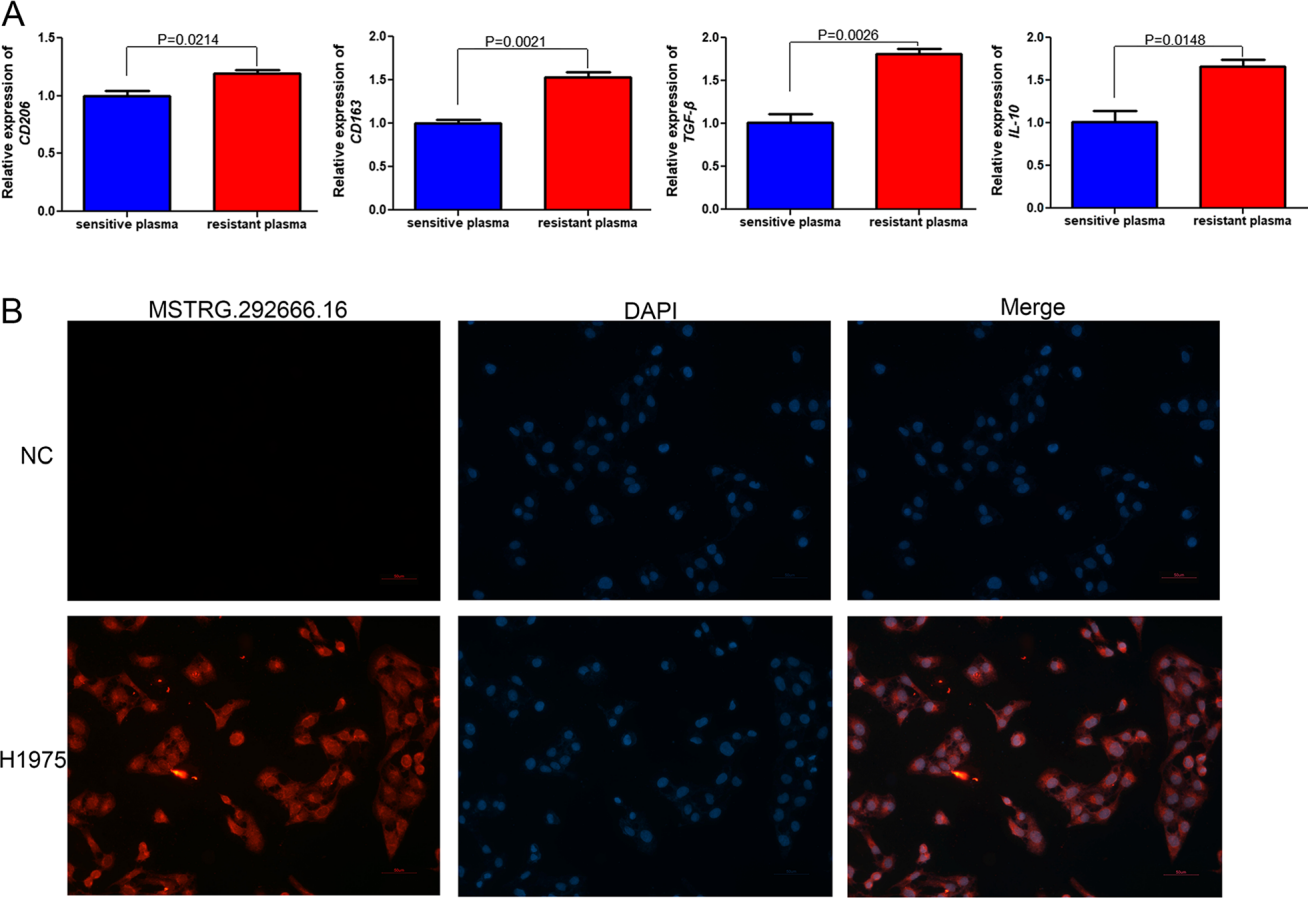
M2 type TAM-related markers in the plasma, and the localization of MSTRG.292666.16 in H1975 cells. **A** The expression levels of *CD206*, *CD163*, *TNF-β*, and *IL-10* were significantly higher in the resistant plasma than those in the sensitive plasma. **B** Fluorescence in situ hybridization showed that lncRNA MSTRG.292666.16 located in the cytoplasm of H1975 cells

To understand the mechanisms by which M2 type TAM-derived exosomes promote drug resistance through lncRNA MSTRG.292666.16, M2 type TAM-related markers were determined in the sensitive and resistant plasma. Compared to the sensitive plasma, the relative expression of *CD206*, *CD163*, *TGF-β*, and *IL-10* in the resistant plasma was markedly upregulated (*P* < 0.05, Fig. 4A). These results showed the existence of M2 type TAM in the osimertinib-resistant plasma. In addition, FISH results showed that lncRNA MSTRG.292666.16 was located in the cytoplasm of H1975 cells (Fig. 4B). Therefore, we further investigated the ceRNA mechanism of M2 type TAM-derived exosomal lncRNA MSTRG.292666.16 in osimertinib resistance in NSCLC in the following study.

### Screening DEmiRNAs in the plasma-derived exosomes and functional analyses

**Fig. 5:**
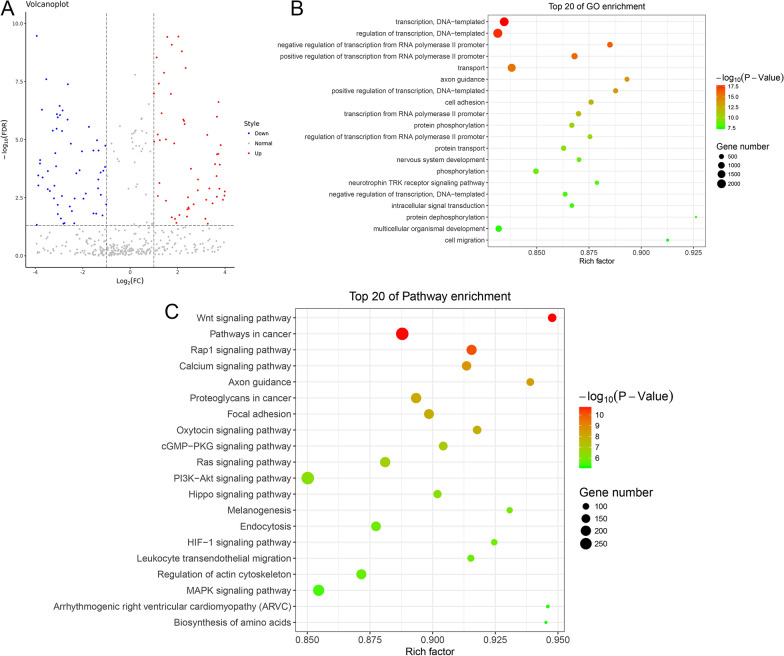
Screen of differential expressed microRNAs (DEmiRNAs) in the plasma-derived exosomes and functional analyses. **A** The volcano figure showed 680 DEmiRNAs, including 393 upregulated miRNAs and 287 downregulated miRNAs, were identified in the resistant plasma-derived exosomes. The blue points represent the downregulated miRNAs; the grey points represent the normal; the red points represented the upregulated miRNAs. **B** The top20 Gene Ontology (GO) terms enriched by the identified DE-miRNAs. **C** The top20 Kyoto Encyclopedia of Genes and Genomes (KEGG) pathways enriched by the identified DE-miRNAs

Exosomes isolated from the sensitive plasma and resistant plasma were identified previously [18] and then sent for miRNA sequencing. Upon sequencing, 680 DEmiRNAs were identified between the exosomes isolated from the sensitive and resistant plasma, including 393 upregulated miRNAs and 287 downregulated miRNAs in the resistant plasma-derived exosomes (Fig. 5A, Additional file 1: Table S1). Then, these DEmiRNAs were subjected to GO terms and KEGG pathway enrichment analysis. It was found that these DEmiRNAs were related to GO terms of “cell adhesion”, “cell migration”, “nervous system development”, “intracellular dephosphorylation” and “neurotrophin TRK receptor signaling pathway” (Fig. 5B). Besides, these DEmiRNAs were significantly enriched in pathways of “Wnt signaling pathway”, “Rap1 signaling pathway”, “Calcium signaling pathway”, “Cgmp-PKG signaling pathway”, “Ras signaling pathway”, “PI3K-Akt signaling pathway”, “HIF-1 signaling pathway” and “MAPK signaling pathway” (Fig. 5C).

### Analysis of ceRNA networks

**Fig. 6:**
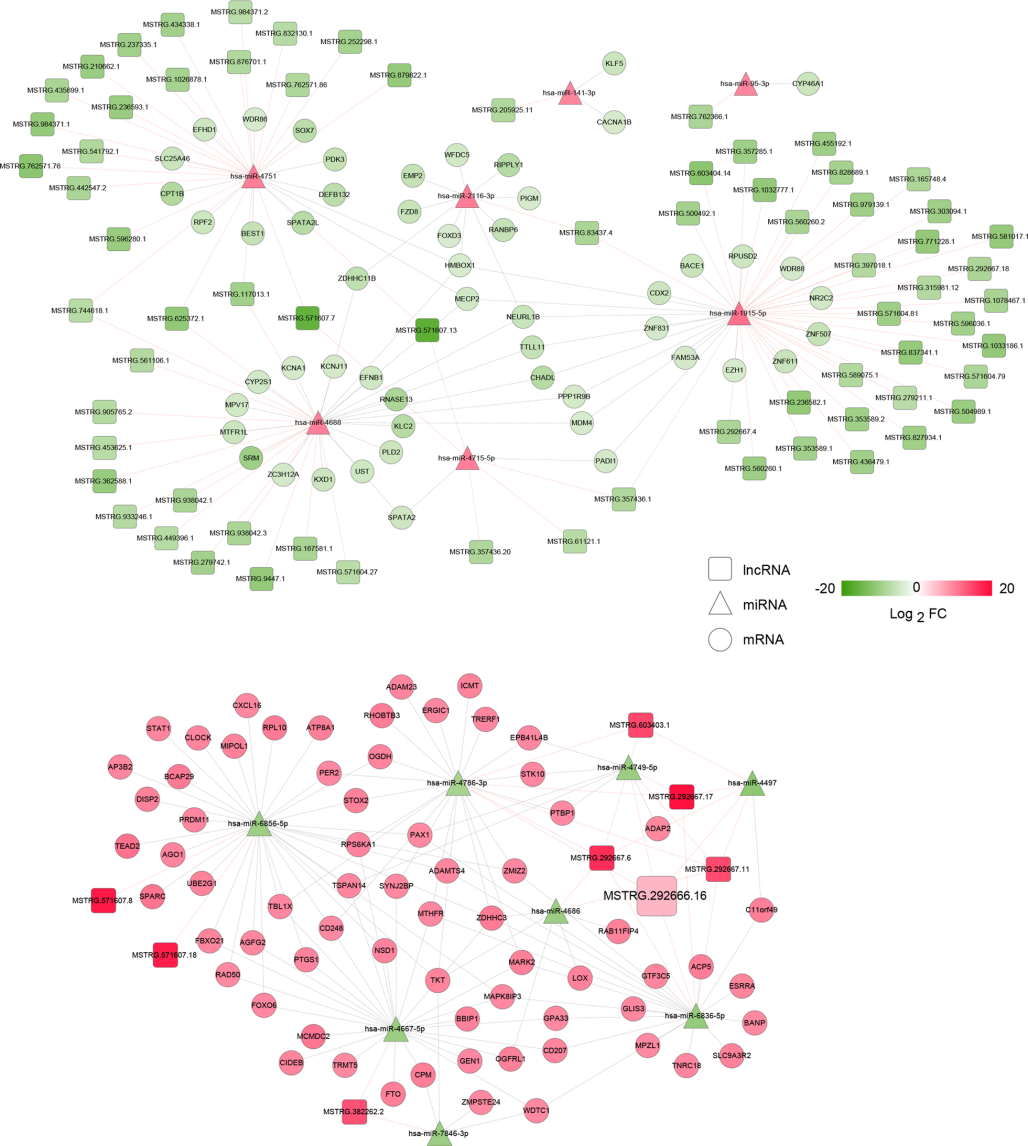
Competing endogenous RNA (ceRNA) networks built by lncRNA-miRNA-mRNA. The squares represent lncRNA; the triangles represent miRNA; and the circles represent mRNA. The node size is associated with the degree. The larger the node size is, the greater the degree, and the more important it is in the networks. Green means the downregulation, and red means upregulation

The analysis of ceRNA network was performed including the previous lncRNA sequencing data from the sensitive and resistant plasma [18]. In total, 103 pairs of DElncRNA-DEmiRNA and 181 pairs of DEmiRNA-DEmRNA were obtained, and ceRNA networks were generated using DElncRNA-DEmiRNA-DEmRNA pairs (Fig. 6). Afterwards, the genes in the ceRNA networks were used for functional analyses. Based on the threshold of *P* < 0.05, 8 GO terms of biology process (“transcription, DNA-templated”, “positive/negative regulation of transcription from RNA polymerase II promoter”, “positive regulation of transcription, DNA-template”, “response to cytokine”, “hippocampus development”, “bone morphogenesis” and “negative regulation of endothelial cell proliferation”) and 7 KEGG pathways (“biosynthesis of antibiotics”, “terpenoid backbone biosynthesis”, “carbon metabolism”, “lysine degradation”, “MAPK signaling pathway”, “serotonergic synapse” and “lysosome”) were significantly enriched (Table 3).

**Table 3:** The enrichment of biological process (BP) gene ontology (GO) terms and Kyoto Encyclopedia of Genes and Genomes (KEGG) in the ceRNA networks

Category	Term	Count	P value	Genes
BP	GO:0000122 ~ negative regulation of transcription from RNA polymerase II promoter	14	9.31E−04	ESRRA, FOXD3, CDX2, STAT1, GLIS3, FZD8, PER2, RIPPLY1, MECP2, KLF5, NSD1, MDM4, TBL1X, WDTC1
	GO:0,006,351 ~ transcription, DNA-templated	25	1.89E−03	GTF3C5, FOXO6, NR2C2, PRDM11, MECP2, HMBOX1, ZNF507, NSD1, ZMIZ2, ZC3H12A, TBL1X, SOX7, TEAD2, BANP, ESRRA, FOXD3, STAT1, TRERF1, PAX1, PER2, RIPPLY1, AGO1, ZNF611, CLOCK, EZH1
	GO:0,045,944 ~ positive regulation of transcription from RNA polymerase II promoter	16	1.99E−03	ESRRA, FOXD3, STAT1, GLIS3, FZD8, NR2C2, PAX1, KLF5, ZMIZ2, AGO1, RPS6KA1, ZC3H12A, TBL1X, CLOCK, EZH1, TEAD2
	GO:0,045,893 ~ positive regulation of transcription, DNA-templated	11	2.18E−03	MECP2, HMBOX1, KLF5, CDX2, STAT1, NSD1, TBL1X, TRERF1, CLOCK, SOX7, BANP
	GO:0,034,097 ~ response to cytokine	4	4.91E−03	SPARC, STAT1, ACP5, CXCL16
	GO:0,021,766 ~ hippocampus development	4	6.04E−03	KCNA1, OGDH, EZH1, PPP1R9B
	GO:0,060,349 ~ bone morphogenesis	3	1.36E−02	RIPPLY1, ACP5, PAX1
	GO:0,001,937 ~ negative regulation of endothelial cell proliferation	3	1.57E−02	SPARC, STAT1, SYNJ2BP
KEGG pathways	hsa01130: Biosynthesis of antibiotics	4	1.11E−02	ICMT, OGDH, TKT, ZMPSTE24
	hsa00900: Terpenoid backbone biosynthesis	2	1.15E−02	ICMT, ZMPSTE24
	hsa01200: Carbon metabolism	3	1.29E−02	OGDH, MTHFR, TKT
	hsa00310: Lysine degradation	2	2.51E−02	NSD1, OGDH
	hsa04010: MAPK signaling pathway	3	4.10E−02	RPS6KA1, CACNA1B, MAPK8IP3
	hsa04726: Serotonergic synapse	2	4.62E−02	CACNA1B, PTGS1
	hsa04142: Lysosome	2	4.91E−02	ACP5, AP3B2

Additionally, it has been reported that the MAPK signaling pathway plays an important role in cancer drug resistance mechanisms [27]. Our study also found that MAPK signaling pathway was closely related to osimertinib resistance of NSCLC, and *RPS6KA1*, *CACNA1B* and *MAPK8IP3* were significantly enriched in “MAPK signaling pathway” (Table 3). Based on the ceRNA networks, we found that lncRNA MSTRG.292666.16 was the hub lncRNA, and *MAPK8IP3* was regulated by lncRNA MSTRG.292666.16/hsa-miR-6836-5p axis (Fig. 6). Therefore, we chose a ceRNA network of lncRNA MSTRG.292666.16-hsa-miR-6836-5p-MAPK8IP3 for further verification.

### ***Validation of lncRNA MSTRG.292666.16-hsa-miR-6836-5p-mRNA-MAPK8IP3 in the exosomes***

**Fig. 7:**
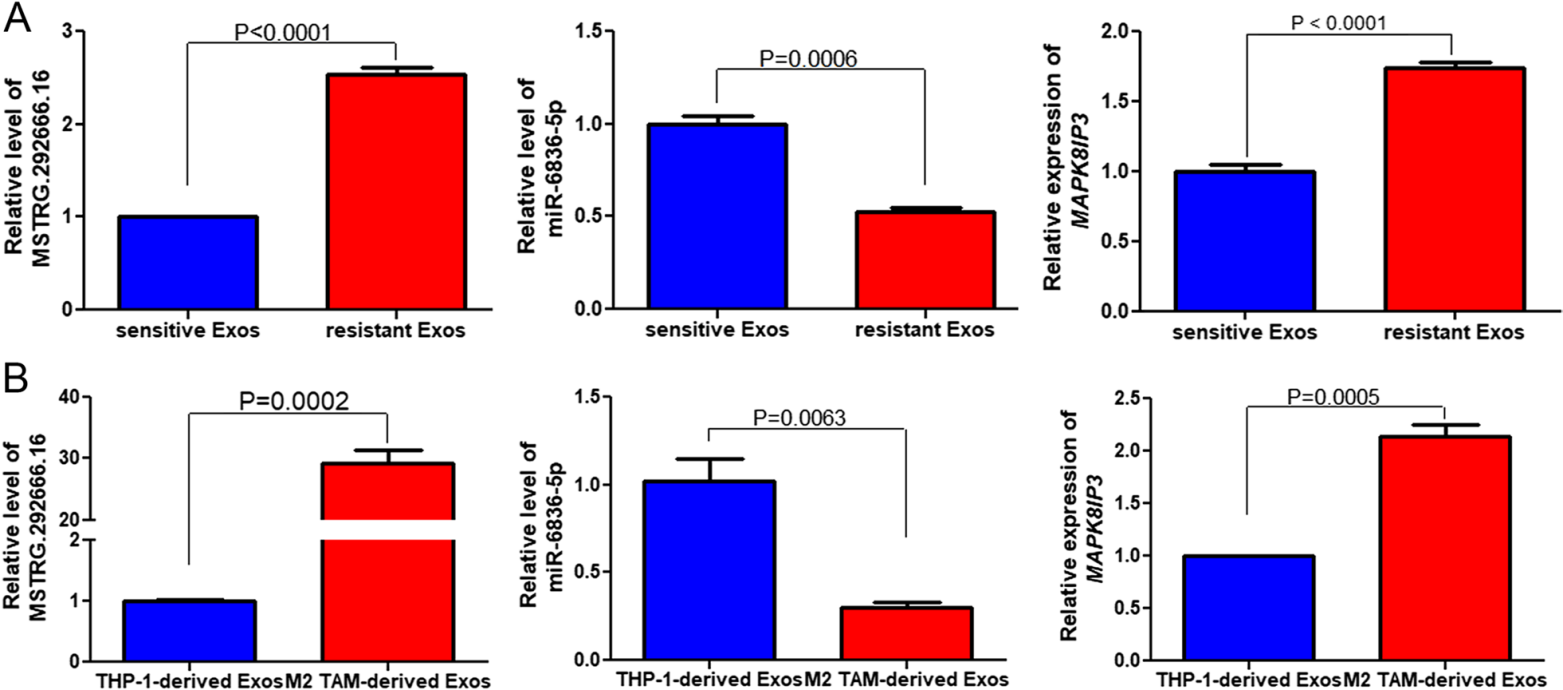
Validation of lncRNA MSTRG.292666.16-hsa-miR-6836-5p-MAPK8IP3 in the exosomes. In the plasma-derived exosomes, lncRNA MSTRG.292666.16, and MAPK8IP3 were upregulated, while hsa-miR-6836-5p was significantly downregulated in the resistant plasma-derived exosomes (**A**). In the cell-derived exosomes, MSTRG.292666.16, and MAPK8IP3 were upregulated, whereas hsa-miR-6836-5p was downregulated in the exosomes from M2 type TAMs (**B**). All these implied that the level trend of MSTRG.292666.16, miR-6836-5p and MAPK8IP3 in the exosomes determined by RT-qPCR were consistent with those measured by sequencing

Compared with the sensitive plasma-derived exosomes, the levels of MSTRG.292666.16 and *MAPK8IP3* were significantly increased in the resistant plasma-derived exosomes (*P* < 0.0001, Fig. 7A, C). The miR-6836-5p level in the exosomes isolated from sensitive and resistant plasma was opposite to that of MSTRG.292666.16 and *MAPK8IP3* levels (Fig. 7B). For the exosomes isolated from the cells, the levels of MSTRG.292666.16 in the THP-1-derived exosomes and M2 type TAM-derived exosomes were 1.00 ± 0.043 and 29.26 ± 3.556, respectively, which showed a significant increase in the M2 type TAM-derived exosomes (*P* = 0.0002, Fig. 7D). A significant reduction in miR-6836-5p level in the M2 type TAM-derived exosomes compared with that in THP-1-derived exosomes was observed (*P* = 0.0063, Fig. 7E). The trend of MAPK8IP3 expression in the exosomes derived from different cells was similar to that of MSTRG.292666.16 (Fig. 7F). The level trends of MSTRG.292666.16, miR-6836-5p, and *MAPK8IP3* in the exosomes determined by RT-qPCR were consistent with those measured by sequencing, which verified the relatively reliable sequencing results.

### ***MSTRG.292666.16 interacted with miR-6836-5p and MAPK8IP3***

**Fig. 8:**
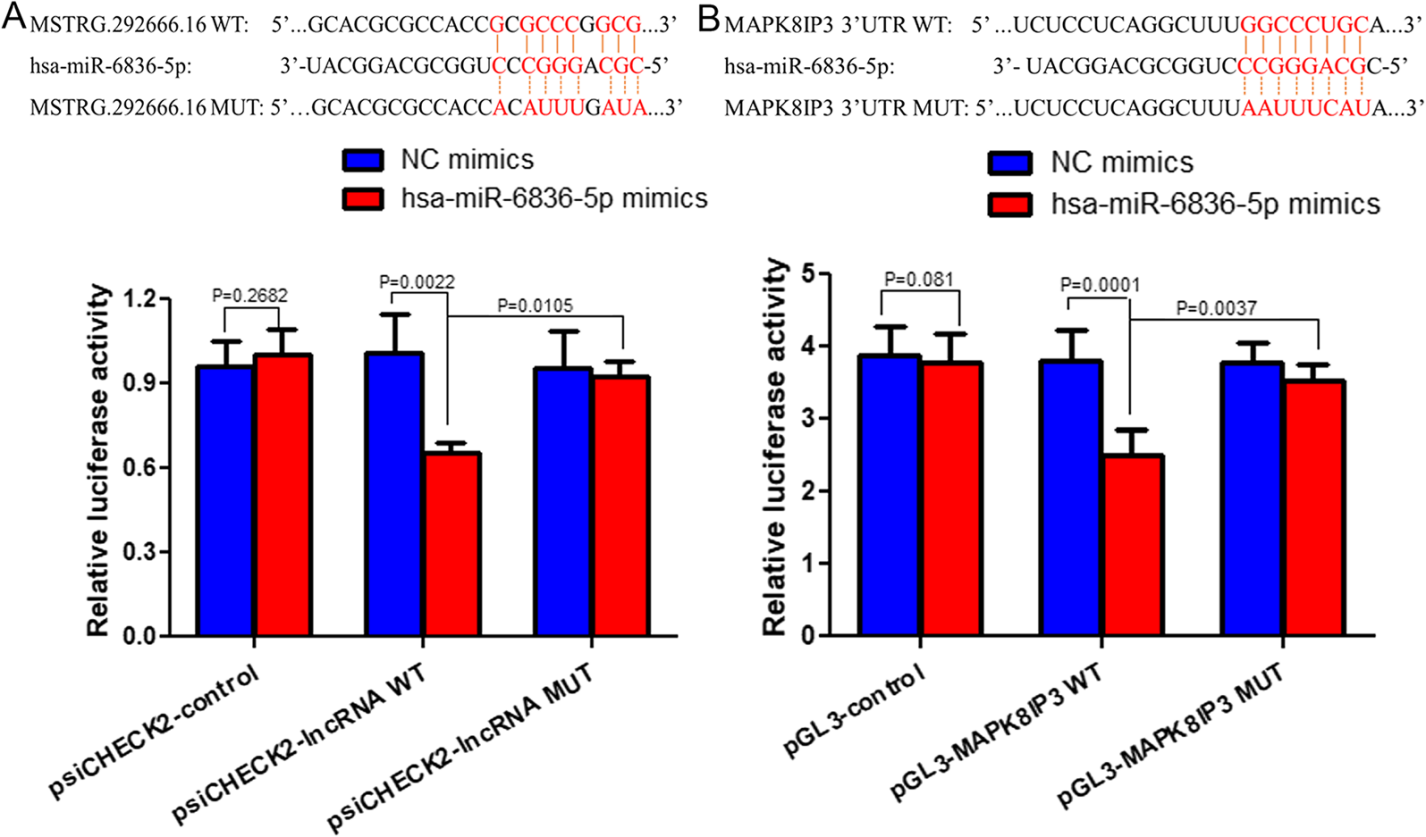
LncRNA MSTRG.292666.16, hsa-miR-6836-5p and mRNA MAPK8IP3 interacted with each other, as indicated by dual-luciferase reporter gene assay. **A** The relationship between MSTRG.292666.16 and miR-6836-5p. **B** The interaction between MAPK8IP3 and miR-6836-5p

Finally, a dual-luciferase reporter gene assay was performed to analyze the relationship between MSTRG.292666.16, miR-6836-5p, and MAPK8IP3. In the control group including psiCHECK2-control and pGL3-control plasmids, no significant differences were observed in luciferase activity between NC mimics and hsa-miR-6836-5p mimics (*P* < 0.05, Fig. 8G, H). In the psiCHECK2-lncRNA WT plasmid, the relative luciferase activity after co-transfected with miR-6836-5p mimics was signally decreased compared with NC mimics (*P* = 0.0022); while in the psiCHECK2-lncRNA MUT plasmid, the luciferase activity was significantly increased (*P* = 0.0105 < 0.05, Fig. 8G). These results indicate that MSTRG.292666.16 is the target of miR-6836-5p. Additionally, in the pGL3-MAPK8IP3 WT plasmid, the relative luciferase activity in the miR-6838-5p mimics group was significantly lower than that in the NC mimics group (*P* = 0.0001); however, after MAPK8IP3 mutation, the relative luciferase activity was significantly enhanced (*P* = 0.0037 < 0.05, Fig. 6H). These results indicate that MAPK8IP3 is the target of miR-6836-5p. Taken together, lncRNA MSTRG.292666.16, hsa-miR-6836-5p, and mRNA MAPK8IP3 could interact with each other.

## Discussion

Lung cancer, especially NSCLC, remains the leading cause of cancer death, and osimertinib is an important first-line drug for EGFR mutation-positive NSCLC [28]. However, with the increasing time of use, the vast majority of patients develop acquired resistance to osimertinib, seriously affecting the survival of patients. Previous studies have reported that acquired EGFR p.T790M (c.2369 C > T; p.Thr790Met) and p.C797S (c.2389 T > A and c.2390G > C, p.Cys797Ser) mutations are closely associated with osimertinib resistance in NSCLC [28, 29]. The EGFR C797S mutation is the most common tertiary EGFR mutation, and cysteine is replaced by serine at codon 797 within the ATP-binding site in this mutation, resulting in the loss of covalent bonds between osimertinib and mutant EGFR [30]. It is predictable that the C797S mutation can also develop cross-resistance to other irreversible EGFR-TKIs by preventing their binding to the EGFR active site [31]. However, the other mechanisms related to osimertinib resistance in NSCLC remain unclear, and novel therapeutic strategies are urgent to be discovered.

Exosomes serve as tools for intercellular communication, and Wu et al. reported that exosomes derived from EGFR wild-type NSCLC could induce osimertinib resistance after being transferred to cancer cells [10]. Our previous study found that osimertinib-resistant exosomes could induce H19575 cells resistant to osimertinib, and exosomal lncRNA MSTRG.292666.16 may be associated with osimertinib resistance in NSCLC [18]. Macrophages can promote important homeostasis functions, such as endocytosis, phagocytosis, and signaling during inflammation [32]. Therefore, this study proposed a hypothesis that lncRNA MSTRG.292666.16 may be transferred from M2 type TAMs of osimertinib-resistant lung cancer, and M2 type TAM-derived exosomes may influence osimertinib resistance in NSCLC through MSTRG.292666.16. CD163 and CD206 are both expressed by macrophages and are markers of macrophage activation [33]. The expression of CD163 can be increased by IL-10 stimulation, and CD206 expression can be upregulated by IL-4 and IL-13 [34]. TGF-β, IL-10, IL-4, and IL-13 are secreted by M2 type macrophages. Besides, M2 type TAMs have been reported to produce Arg-1 and promote wound healing by increasing the generation of Arg-1 and pro-chondrogenic cytokines (IL-10 and TGF-β) [35]. Our study determined the expression of CD163, CD206, TGF-β, IL-10, and Arg-1 in the plasma of NSCLC patients and found that their expression was significantly upregulated in osimertinib-resistant plasma, which indicated the existence of M2 type TAMs in osimertinib-resistant plasma.

Thereafter, M2 type TAMs were successfully established, and exosomes were isolated from these cells. Tumor-forming experiments in nude mice showed that M2 type TAM-derived exosomes and MSTRG.292666.16 overexpression could enhance osimertinib resistance in NSCLC. A previous study demonstrated that lncRNA ZFAS1 was highly expressed in the tissues of esophageal squamous cell carcinoma, and exosomes with ZFAS1 overexpression significantly promoted cell-based xenograft tumor growth [36]. Another study by Qu et al. showed that high levels of lncRNA ARSR were observed in sunitinib-resistant renal cell carcinoma cells, and locked nucleic acid (LNA)-mediated ARSR knockdown effectively restored the sensitivity of xenografts to synchronous sunitinib therapy [37]. Furthermore, our results also showed that the MSTRG.292666.16 level was significantly higher in the M2 type TAM-derived exosomes than in the THP-1-derived exosomes. Together with our results, it can be inferred that M2 type TAM-derived exosomes may improve osimertinib resistance in NSCLC by upregulating MSTRG.292666.16.

Previous studies have shown that ceRNAs influence the progression of liver cancer, and cytoplasmic lncRNAs can function as ceRNAs regulating mRNA stabilization or translation by sponging miRNAs, and thus affecting signaling pathways [38, 39]. In this study, FISH results showed that lncRNA MSTRG.292666.16 localized in the cytoplasm of H1975 cells, which suggested its potential involvement in ceRNA mechanisms. Small sequencing identified 680 DEmiRNAs, including 393 upregulation and 287 downregulation in exosomes from resistant plasma. Then, by combining the miRNA sequencing data with our previous lncRNA sequencing data, ceRNA networks were constructed, and generated 103 pairs of DElncRNAs-DEmiRNAs and 181 pairs of DEmiRNAs-DEmRNAs. The genes in the ceRNA networks were significantly enriched in 8 GO terms and 7 KEGG pathways, such as “biosynthesis of antibiotics” (*ICMT*, *OGDH*, *TKT*, *ZMPSTE24*), “terpenoid backbone biosynthesis” (*ICMT*, *ZMPSTE24*), and “MAPK signaling pathway” (*RPS6KA1*, *CACNA1B*, *MAPK8IP3*). *ICMT* has been reported to facilitate the growth, migration, and doxorubicin resistance of hepatocellular carcinoma via various carcinogenic pathways [40]. *OGDH*, a subunit of the OGDH complex, is involved in the TCA cycle, energy balance, and cellular signaling. Lu et al. [41] showed that *OGDH*, which serves as a positive regulator of gastric cancer progression, enhances mitochondrial function, and activates the Wnt/β-catenin signaling pathway. TKT was upregulated in the NSCLC cells, and its knockdown strengthened the action of gefitinib in NSCLC cells [42]. Loss of *ZMPSTE24* accelerates senescence associated with cancer [43]. The MAPK signaling pathway has been shown to be activated in many cancers that are resistant to drugs [27], and our study found that *RPS6KA1*, *CACNA1B*, and *MAPK8IP3* were significantly enriched in the MAPK signaling pathway. Fan et al. [44] demonstrated that the octreotide-paclitaxel conjugate could reverse paclitaxel resistance in A2780/Taxol human ovarian cancer cells by repressing the activity of the MAPK signaling pathway. These reports together with our results support that *ICMT*, *OGDH*, *TKT*, *ZMPSTE24*, *RPS6KA1*, *CACNA1B*, and *MAPK8IP3*, as well as pathways for biosynthesis of antibiotics, terpenoid backbone biosynthesis, and MAPK signaling pathway may play important roles in osimertinib resistance in NSCLC.

The MAPK signaling pathway has been reported to be activated in cancer drug resistant samples, and its abnormal activation could contribute to cell loss of differentiation and apoptosis [27], and could be a mechanism of resistance to osimertinib in EGFR-mutated NSCLC [28]. Our established ceRNA networks demonstrated that *RPS6KA1*, *CACNA1B* and *MAPK8IP3* were significantly enriched in “MAPK signaling pathway”, and lncRNA MSTRG.292666.16 was the hub node. Therefore, a ceRNA network of lncRNA MSTRG.292666.16- miR-6836-5p-MAPK8IP3 was chosen for further validation. The levels of MSTRG.292666.16 and MAPK8IP3 were significantly increased in resistant plasma-derived exosomes and M2 type TAM-derived exosomes, while miR-6836-5p levels were significantly evidently reduced, which was consistent with the expression trend of ceRNA mechanisms. The dual-luciferase reporter gene assay indicated that MSTRG.292666.16, miR-6836-5p, and MAPK8IP3 could interact with each other. A previous study reported that HOTAIR was the target of miR-331-3p, and HER2 was the target of miR-331-3p through a luciferase assay, which suggested that lncRNA HOTAIR could regulate *HER2* expression by sponging miR-331-3p in gastric cancer [45]. Another study found that lncRNA UCA1, miR-143, and FOSL2 interacted with each other in a luciferase reporter assay, and UCA1 packaged into exosomes could promote gefitinib resistance in NSCLC cells by mediating miR-143/FOSL2 [46]. Therefore, we speculated that M2 type TAM-derived exosomes may enhance osimertinib resistance in NSCLC by regulating the MSTRG.292666.16/miR-6386-5p/MAPK8IP3 axis.

However, this study has some limitations. First, the specific ceRNA mechanism of MSTRG.292666.16/miR-6836-5p/MAPK8IP3 through M2 type TAM-derived exosomes in osimertinib-resistant NSCLC needs to be further investigated in vitro. Moreover, a larger sample size is needed to verify our conclusions, and further experiments should be conducted to investigate the effects of the identified genes and pathways on osimertinib resistance in NSCLC.

## Conclusions

In conclusion, through sequencing, it was found that *ICMT*, *OGDH*, *TKT*, *ZMPSTE24*, *RPS6KA1*, *CACNA1B*, and *MAPK8IP3*, as well as pathways for biosynthesis of antibiotics, terpenoid backbone biosynthesis, and MAPK signaling pathway may play important roles in osimertinib resistance in NSCLC. Additionally, M2 type TAM-derived exosomes may promote osimertinib resistance in NSCLC via the MSTRG.292666.16/miR-6836-5p/MAPK8IP3 axis. Our work illuminated a novel mechanism of osimertinib resistance in NSCLC, and implied that M2 type TAM-derived exosomal MSTRG.292666.16/ miR-6836-5p/ MAPK8IP3 serves as a novel therapeutic target and may have potential therapeutic value in treating osimertinib resistance in NSCLC.

## Supplementary Information

**Additional file 1: Table S1.** All differentially expressed miRNAs between the exosomes isolated from the sensitive and resistant plasma with the thresholds of |log_2_Fold change (FC)|> 1 and false discovery rate (FDR) < 0.05.

## Data Availability

The dataset used and/or analyzed during the current study are available from the corresponding author on reasonable request.
